# Association Between Myocardial Dysfunction and Septic Shock

**DOI:** 10.3390/ijms27062552

**Published:** 2026-03-10

**Authors:** Vlad Pădureanu, Daniel Cosmin Caragea, Denisa Floriana Vasilica Pîrșcoveanu, Dalia Dop, Alexandru Claudiu Munteanu, Dumitru Rădulescu, Dragoș George Popa, Dragoș Forțofoiu, Alice Nicoleta Drăgoescu, Rodica Pădureanu

**Affiliations:** 1 Department of Internal Medicine, University of Medicine and Pharmacy of Craiova, 200349 Craiova, Romania; vlad.padureanu@umfcv.ro (V.P.); dragos.fortofoiu@umfcv.ro (D.F.); rodica.padureanu@umfcv.ro (R.P.); 2 Department of Nephrology, University of Medicine and Pharmacy of Craiova, 200349 Craiova, Romania; daniel.caragea@umfcv.ro; 3 Department of Neurology, University of Medicine and Pharmacy of Craiova, 200349 Craiova, Romania; denisa.pirscoveanu@umfcv.ro; 4 Department of Pediatrics, University of Medicine and Pharmacy of Craiova, 200349 Craiova, Romania; dalia.dop@umfcv.ro; 5 Department of Surgery, University of Medicine and Pharmacy of Craiova, 200349 Craiova, Romania; 6 Department of Plastic Surgery, University of Medicine and Pharmacy of Craiova, 200349 Craiova, Romania; dragos.popa@umfcv.ro; 7 Department of Anesthesiology and Intensive Care, University of Medicine and Pharmacy of Craiova, 200349 Craiova, Romania; alice.dragoescu@umfcv.ro

**Keywords:** sepsis, septic shock, myocardial dysfunction, cardiomyopathy

## Abstract

There is a substantial correlation between cardiac dysfunction and elevated mortality in sepsis. Impaired myocardial perfusion, direct myocardial injury, and mitochondrial dysfunction are all part of the complex pathophysiology of sepsis-induced myocardial dysfunction. Recent evidence has shown the critical role mitochondrial dysfunction plays in the development of sepsis-induced myocardial dysfunction. In order to prevent and treat sepsis-induced myocardial dysfunction, a variety of drugs have been proposed. However, patient outcomes have not been appreciably enhanced by this therapy. This underscores the need for novel treatment approaches that target the specific pathways underlying cardiac dysfunction in sepsis. The prognosis is greatly impacted by sepsis-induced cardiac dysfunction, monitoring it is crucial. Clinicians employ a mix of clinical evaluations, hemodynamic monitoring, echocardiography, and bSICiomarkers to efficiently monitor this illness. The combined application of these techniques provides a comprehensive evaluation of cardiac function, thereby supporting timely optimization of treatment strategies. Treatments for septic shock and established sepsis will be beneficial for patients with this condition. However, there is little information and evidence about more targeted therapy, except than general management with vasopressors, inotropes, and fluid resuscitation. This study provides an outline of current knowledge on the pathophysiological mechanisms underlying sepsis-induced cardiac dysfunction, as well as the effects of monitoring and current treatments on sepsis-induced myocardial dysfunction.

## 1. Introduction

Sepsis remains a major burden in intensive care units (ICUs) and is associated with high in-hospital mortality, particularly among critically ill patients, despite advances in supportive care, antimicrobial therapy, and early recognition. Sepsis is a systemic inflammatory response syndrome that arises from a dysregulated immune response to pathogens infection leading to potentially life-threatening organ dysfunction [1].

Cardiovascular involvement is a major consequence of sepsis and has a significant impact on patient outcomes [2]. Sepsis-related myocardial dysfunction encompasses a spectrum of acute cardiac abnormalities and has been the subject of extensive investigation over the past five decades [3]. This condition is associated with a markedly increased mortality rate of 20–50% [4,5].

Recent evidence suggests that myocardial dysfunction during septic shock should not be considered a single homogeneous entity but rather a dynamic spectrum of cardiovascular phenotypes. Clinical manifestations may range from isolated left ventricular systolic impairment to predominant right ventricular dysfunction, diastolic abnormalities with preserved ejection fraction, or hyperdynamic states characterized by impaired myocardial efficiency. This heterogeneity likely reflects differences in host immune response, microcirculatory alterations, mitochondrial resilience, and underlying cardiovascular reserve, with important implications for prognosis and therapeutic strategies. In the literature, terms such as sepsis-induced cardiomyopathy, septic cardiomyopathy, and sepsis-induced myocardial dysfunction (SIMD) are commonly used to describe these sepsis-related cardiac alterations [6], often encompassing this broader and evolving phenotypic spectrum. For clarity and consistency throughout this review, we will refer to this condition using the term sepsis-induced myocardial dysfunction (SIMD).

Sepsis-induced myocardial dysfunction (SIMD), a reversible impairment of cardiac contractility and relaxation that occurs in the context of systemic inflammation, circulatory dysregulation, and cellular metabolic derangements, is one of the most well-known cardiovascular manifestations of sepsis [7,8]. SIMD has been linked to hemodynamic instability, higher vasopressor requirements, and worse clinical outcomes. Depending on the diagnostic methods and definitions employed, its prevalence was reported from 40% to 60% of patients with septic shock [9].

Sepsis-induced myocardial dysfunction is characterized by a multifaceted pathophysiology that includes inflammatory cytokine release, mitochondrial impairment, microvascular dysfunction, and abnormal calcium homeostasis [10]. Assessment of clinical severity relies on echocardiography, invasive and noninvasive hemodynamic monitoring, and biomarkers, including cardiac troponins and natriuretic peptides. Persistent dysfunction has been linked to higher mortality, longer ICU stays, and a larger requirement for circulatory support, even though SIMD is frequently temporary and reversible within seven to ten days in survivors [11,12]. These temporal dynamics imply that the presence and resolution of SIMD across time may both have an impact on its predictive relevance.

Despite extensive research into its pathophysiology, clinical manifestations, and diagnostic approaches, SMID remains a challenging condition to diagnose. At present, no definitive therapeutic strategies are available, and significant gaps in understanding persist. Recent investigations have focused on characterizing the patterns of myocardial dysfunction in sepsis and elucidating their underlying mechanisms. Evidence suggests that an impaired host immune response to invasive pathogens contributes to sepsis-related cardiovascular injury, triggering a systemic inflammatory cascade amplified by circulating inflammatory mediators. These processes lead to hemodynamic alterations and subsequent impairment of cardiac function [13,14]. Despite substantial research into numerous biochemical mediators and molecular pathways, the precise mechanisms responsible for myocardial depression in sepsis remain incompletely understood. This review aims to summarize current insights into the pathophysiological mechanisms and clinical manifestations of SIMD, with a particular focus on monitoring strategies and available therapeutic approaches.

## 2. Pathophysiological and Clinical Mechanisms of Sepsis-Induced Myocardial Dysfunction

**Figure 1:**
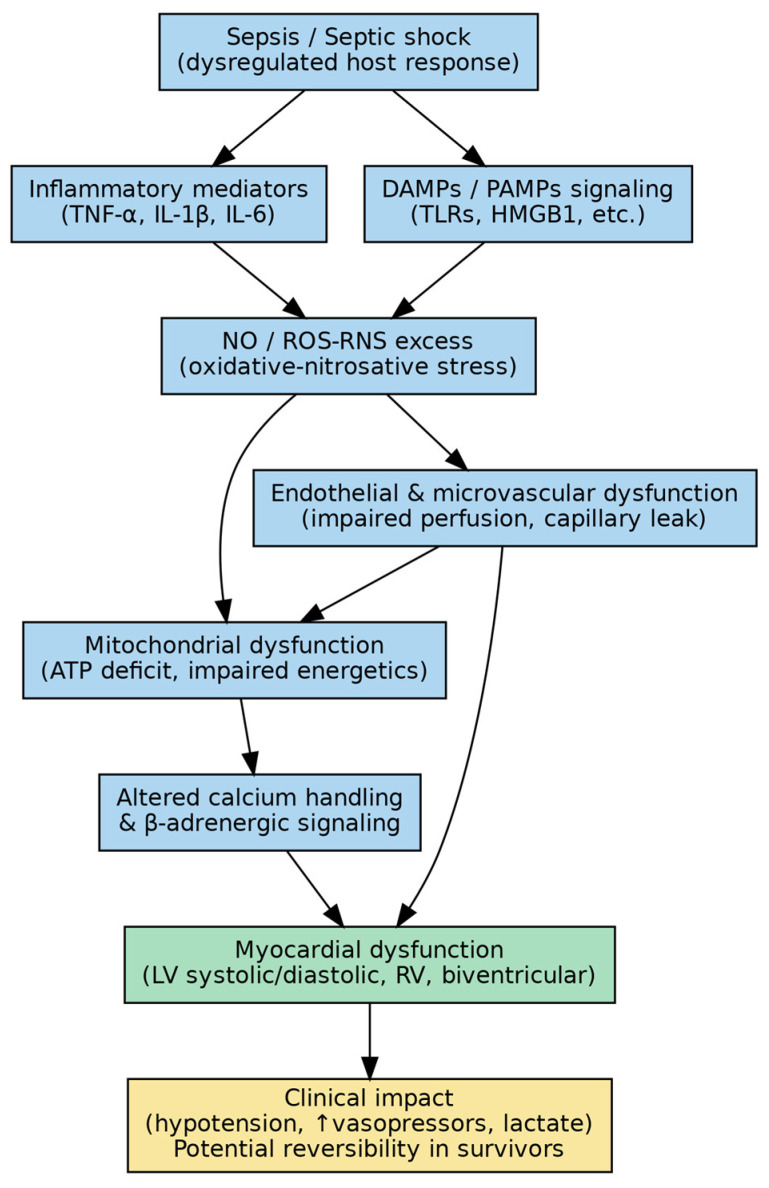
Proposed mechanisms linking septic shock to myocardial dysfunction.

**Figure 2:**
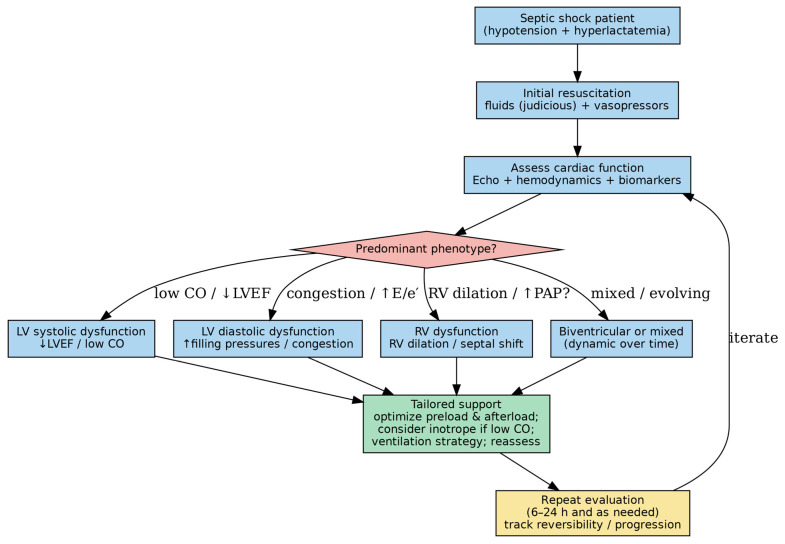
Bedside conceptual approach to identify septic shock-related myocardial dysfunction phenotypes and guide supportive management.

The key clinical phenotypes and the main mechanistic pathways and management implications are summarized in Figure 1 and Figure 2.

Figure 1 illustrates the interconnected mechanisms linking septic shock to myocardial dysfunction. Rather than representing isolated pathways, the depicted processes interact dynamically. Systemic inflammation triggered by PAMPs and DAMPs activates Toll-like receptor signaling, leading to cytokine release, nitric oxide overproduction, and endothelial injury. These alterations promote microcirculatory dysfunction and impair myocardial oxygen utilization despite preserved or even increased coronary blood flow. Concurrent mitochondrial injury and oxidative stress reduce ATP generation and alter calcium handling, culminating in impaired contractility and diastolic dysfunction. The schematic emphasizes that septic cardiomyopathy results from the convergence of inflammatory, metabolic, and neurohumoral disturbances rather than a single dominant mechanism.

Septic shock triggers an inflammatory and neurohumoral cascade that promotes endothelial and microcirculatory dysfunction, mitochondrial impairment and energetic failure, oxidative/nitrosative stress, and altered β-adrenergic signaling, culminating in reduced myocardial contractility and impaired relaxation, with possible LV and/or RV involvement.

Abbreviations: ATP, adenosine triphosphate; DAMPs, damage-associated molecular patterns; HMGB1, high mobility group box 1; IL-1β, interleukin-1 beta; IL-6, interleukin-6; LV, left ventricle; NO, nitric oxide; PAMPs, pathogen-associated molecular patterns; RNS, reactive nitrogen species; ROS, reactive oxygen species; RV, right ventricle; TLRs, Toll-like receptors; TNF-α, tumor necrosis factor alpha.

A pragmatic scheme integrating echocardiography and hemodynamic assessment to screen for LV systolic/diastolic dysfunction and RV involvement; findings may support phenotype-oriented optimization of preload, vasopressors/inotropes, and ventilation, with iterative reassessment.

Figure 2 categorizes septic myocardial dysfunction into recognizable clinical phenotypes, the mechanisms underlying this phenotypic heterogeneity remain incompletely understood. The same septic insult may lead to divergent functional patterns—ranging from isolated LV systolic dysfunction to predominant RV impairment or preserved ejection fraction with diastolic abnormalities reflecting variability in host immune responses, preexisting cardiovascular reserve, microvascular reactivity, and mitochondrial resilience. Differential activation of inflammatory signaling pathways, variability in nitric oxide production, heterogeneity in mitochondrial bioenergetic capacity, and distinct patterns of regulated cell death (apoptosis, necroptosis, or ferroptosis) may contribute to these divergent phenotypes.

Abbreviations: CO, cardiac output; E/e′, ratio of early transmitral inflow velocity (E) to early diastolic mitral annular tissue velocity (e′), a surrogate of LV filling pressures; LVEF, left ventricular ejection fraction; PAP (sPAP), (estimated systolic) pulmonary artery pressure; RV, right ventricle.

### 2.1. Clinical Signs of Myocardial Dysfunction Brought on by Sepsis

Biventricular dilatation, a reversible decrease in ejection fraction, a reduced blood pressure response to intravenous fluid resuscitation, and an inability to increase cardiac output despite elevated levels of circulating catecholamines are all indicators of contractile dysfunction, a major characteristic of cardiac impairment in sepsis [3]. Clinically, myocardial dysfunction may manifest as dilatation of both ventricles, right ventricular (RV) impairment, left ventricular (LV) systolic dysfunction, or LV diastolic dysfunction. Significant afterload reduction connected to lower cardiac output/cardiac index and systemic vascular resistance (SVR) has been linked to pump failure in studies. This results in regional or global contractile dysfunction. Generally speaking, LV and RV dilatation is an adaptive response that frequently results from improved compliance. However, right ventricular dysfunction adversely affects left ventricular function by reducing left ventricular ejection fraction, increasing left ventricular filling pressures, and causing mechanical compromise through interventricular septal displacement [15,16].

In the past, it was thought that cardiac dysfunction only happened during the hypodynamic phase of shock, which is characterized by elevated SVR, tissue hypoperfusion, and ultimately organ failure. However, it is now known that hyperdynamic septic shock, which is characterized by increased cardiac output, lower SVR, and warm, well-perfused skin, can also cause myocardial damage [17]. LV dilatation is thought to be an adaptive mechanism in hyperdynamic septic shock that aids in maintaining cardiac output through the Starling mechanism. This adaptation is linked to improved prognosis and reduced mortality in sepsis patients. A spectrum of isolated diastolic dysfunction and a combination of both systolic and diastolic abnormalities have been identified as part of the pathophysiology of LV systolic failure in septic individuals. In comparison to controls, septic patients were shown to have an elevated LV end-diastolic volume index, indicating acute LV dilatation and a lower LV ejection fraction [18,19,20].

Right ventricular systolic failure is characterized by RV dilatation and reduced RV ejection fraction. Evidence suggests that increased RV afterload is unlikely to be the primary mechanism underlying RV depression in septic shock, as RV dysfunction occurs independently of changes in pulmonary arterial pressure and systemic vascular resistance. Notably, although sepsis-related RV dilatation can lead to interventricular septal displacement—thereby reducing LV compliance, preload, and overall systolic performance, this phenomenon remains underreported in the literature [21]. Recent research suggests that sepsis-induced right ventricular (RV) dysfunction is often similar to left ventricular (LV) dysfunction, despite the anatomical and functional distinctions between the ventricles.

### 2.2. Risk Factors for Cardiac Dysfunction Brought on by Sepsis

Myocardial dysfunction can arise in the setting of sepsis due to a number of risk factors:

1. Sepsis severity: Myocardial dysfunction is more likely in cases of severe sepsis, such as septic shock. Widespread inflammation and severe infections can have a major effect on heart function.
2. Preexisting cardiovascular disease: Patients with pre-existing cardiovascular conditions—such as hypertension, coronary artery disease, or heart failure—are more susceptible to sepsis-induced myocardial dysfunction, as these comorbidities may limit the heart’s ability to tolerate the additional hemodynamic and inflammatory stress imposed by sepsis.
3. Associated comorbidities: Because they affect general health and the inflammatory response, illnesses like diabetes mellitus or chronic obstructive pulmonary disease (COPD) can increase the risk.
4. High levels of inflammatory markers: Elevated levels of inflammatory markers, particularly pro-inflammatory cytokines, may exacerbate cardiac dysfunction and are frequently observed in severe sepsis.
5. High acute illness severity: Myocardial dysfunction is more likely to occur in patients who have severe organ dysfunction and high Sequential Organ Failure Assessment (SOFA) scores.
6. Nutritional status: Inadequate nutrition can weaken the immune system and make a person more vulnerable to severe sepsis and cardiac abnormalities.
7. Inadequate or delayed treatment: More serious systemic effects, such as heart malfunction, might result from inadequate management of sepsis or from delays in treating it.
8. High lactic acid: SIMD is independently associated with elevated serum lactate levels (>4.0 mmol/L) at ICU admission [22,23,24]. Accordingly, early identification of at-risk patients, prompt initiation of appropriate therapy, and optimized supportive care are essential to mitigate the impact of sepsis-induced myocardial dysfunction.

### 2.3. Pathophysiology of Cardiac Dysfunction Caused by Sepsis

Understanding sepsis-induced myocardial dysfunction is closely intertwined with our understanding of sepsis itself. The pathophysiology of sepsis involves an uncontrolled inflammatory response resulting from dysregulated interactions between the host immune system and invasive microorganisms. Pattern recognition receptors (PRRs), which attach to pathogen-associated molecular patterns (PAMPs), are often how the host immune system identifies pathogenic invaders. Immune cells have PRRs, such as Toll-like receptors (TLRs), on their surface, whereas invasive bacteria include PAMPs, such as lipopolysaccharide (LPS) and lipoteichoic acid. By identifying and attaching to danger-associated molecular patterns (DAMPs) generated during inflammation, PRRs not only trigger immune responses against pathogens but also activate the innate immune system [25]. Ca5, complement a5; NO, nitric oxide; PAMPs, pathogen-associated molecular patterns; DAMPs, damage-associated molecular patterns; and ROS, reactive oxygen species.

The pathogenesis of SIMD is still poorly understood despite decades of investigation. The body’s reaction to surgical, traumatic, ischemic, or septic insults causes the release of cytokines, which have recently been identified as the main mediators of myocardial depression in sepsis [26]. But SIMD can also be caused by a number of different reasons, which emphasizes the necessity of looking into certain contributing pathways in order to create therapeutic targets that work.

#### 2.3.1. Myocardial Circulation

One of the key mechanisms underlying sepsis-induced myocardial dysfunction is reduced myocardial perfusion. The concept of myocardial hibernation—an adaptive response to ischemia and hypoxia—has been proposed to explain the reversible nature of myocardial depression observed in sepsis. In sepsis, this behavior is thought to be a typical sign of cardiac dysfunction [27]. In patients with severe sepsis, ischemia of the heart muscle can considerably exacerbate septic myocardial dysfunction despite this preventive mechanism. The intravascular fluid state has a significant impact on cardiac function. One of the main causes of hemodynamic instability in septic patients is the loss of vascular tone brought on by arterial dilatation. Cardiac contractility, end-systolic ventricular elastance (Ees), arterial elastance (Ea), and left ventricular (LV) systolic performance are physiological parameters that have recently been discovered. Ventriculo-arterial coupling, or the ratio of Ea to Ees, is another important metric for evaluating the cardiovascular system’s effectiveness. Although the endothelium is important in sepsis, little is known about the consequences of endothelial dysfunction linked to sepsis. According to a prior study, optimum coronary flow did not stop myocardial dysfunction in individuals with septic shock, even if it maintained or even increased microcirculatory coronary blood flow. This was explained by blood flow maldistribution caused by septic endothelium injury. The idea that there are several contributing causes to SIMD is supported by this data [28]. There is still little evidence to support the idea that myocardial ischemia alone is the main source of myocardial dysfunction in sepsis, despite the fact that these studies show notable alterations in coronary flow and myocardial metabolism.

#### 2.3.2. Dysfunction of Mitochondrial Metabolism

Numerous studies have demonstrated that mitochondrial dysfunction is a key contributor to SIMD, primarily through impaired energy production, although the precise mechanisms underlying mitochondrial failure in sepsis remain incompletely understood. Given the heart’s high mitochondrial density and dependence on oxidative metabolism, mitochondrial dysfunction is closely associated with the severity and prognosis of sepsis-induced cardiac dysfunction. Because the heart depends heavily on adenosine triphosphate (ATP) to sustain contraction and diastolic function, it is especially vulnerable to sepsis-induced mitochondrial dysfunction. Numerous methods, most notably mitochondrial uncoupling proteins found in the inner mitochondrial membrane, have been studied to restore mitochondrial function. These proteins enable proton leakage across the membrane, which is essential for controlling the potential of the mitochondrial membrane and producing ATP and reactive oxygen species (ROS). Oxidative stress, mitochondrial Ca^2+^ flux, mitochondrial DNA (mtDNA) in sepsis, mitochondrial dynamics, mitochondrial biogenesis, and mitochondrial autophagy are additional pertinent biochemical processes [29,30,31].

##### Oxidative Stress and Damage to Mitochondria

Elevated amounts of reactive oxygen species (ROS), including superoxide, are caused by mitochondrial dysfunction in sepsis, which exacerbates oxidative injury and prolongs damage to cells and organs [32]. In the septic myocardium, an imbalance in ROS and reactive nitrogen species interferes with oxidative phosphorylation, which directly prevents mitochondrial respiration and damages a number of subcellular components, including mitochondrial proteins, which frequently leads to mitochondrial death. This oxidative stress has been seen in septic patients and is strongly associated with organ failure and mitochondrial dysfunction in sepsis models [33,34]. Furthermore, because oxidative stress lowers levels of antioxidants including uric acid, unconjugated bilirubin, and vitamins C and E, it weakens the antioxidant ability. Antioxidants may therefore present a promising treatment path in the future, as oxidative stress is a key factor in septic cardiac dysfunction [35,36,37].

##### Abnormal Calcium Transport in the Mitochondria

The creation of a proton gradient, which depends on the impermeability of the inner mitochondrial membrane and is mostly controlled by Ca^2+^, is a crucial stage in the synthesis of ATP [38,39]. By encouraging its escape from the sarcoplasmic reticulum and decreasing its absorption, the release of inflammatory mediators, such as cytokines, during sepsis raises mitochondrial Ca^2+^ levels [40]. In addition to causing irreversible mitochondrial damage and ultimately impairing cardiomyocyte contractile performance, this Ca^2+^ overload triggers the opening of mitochondrial permeability transition pores (mPTPs) and sets off a chain of pathological alterations, including caspase activation [41,42]. Vasodilation follows smooth muscle relaxation when cytoplasmic Ca^2+^ levels fall. This decrease in Ca^2+^ levels in heart cells leads to impaired myocardial function and decreased myocyte contraction [43]. A number of routes have been linked to septic myocardial depression, including decreased sensitivity of Ca^2+^ channels, altered Ca^2+^ release from the sarcoplasmic reticulum, and impaired Ca^2+^ uptake, given the critical function of Ca^2+^ in cardiac contraction [44]. Therefore, one important factor affecting heart function is aberrant Ca^2+^ transport.

##### DNA in Mitochondria

Damage to mitochondrial DNA (mtDNA), which is implicated in the immune system and can serve as a biomarker to predict mortality in septic patients due to its elevated plasma levels, is also associated with mitochondrial dysfunction during sepsis. mtDNA can leak into the cytosol during sepsis due to increased ROS generation, decreased mitochondrial membrane potential (MMP), and the opening of the mitochondrial permeability transition pore (mPTP). The release of mtDNA can activate Toll-like receptors, which in turn trigger the production of IL-1β and IL-18, ultimately leading to cell death, since mtDNA functions as a Damage-Associated Molecular Pattern (DAMP). During sepsis, mtDNA is more vulnerable to destruction. This damage triggers apoptosis, initiates calcium-dependent stress signaling, impairs oxidative phosphorylation, and triggers inflammatory responses. Circulating mtDNA levels are significantly greater in non-survivors than in survivors, according to studies involving patients in intensive care units. These results imply that worse outcomes for sepsis patients are associated with higher mtDNA concentrations [45,46,47].

##### Dynamics of Mitochondria

To control their size, quantity, and shape, mitochondria undergo cycles of fission and fusion. This equilibrium between mitochondrial fusion and fission is essential for preserving cellular and mitochondrial homeostasis under normal physiological settings. This includes controlling cell division, proliferation, and the removal of damaged mitochondria. Proteins including mitofusin-1 (encoded by MFN1) and mitofusin-2 (encoded by MFN2) are involved in mitochondrial fusion, whereas the dynamin-like GTPase (encoded by OPA1) mediates mitochondrial fission [48]. Reduced oxygen consumption and alterations in mitochondrial membrane potential are associated with these proteins. Fission and fusion rates rise in response to stress, and these activities are essential for removing damaged mitochondria and promoting healing. Despite their significance, little is known about the specific functions of these proteins in fission and fusion. Organ failure results from fragmented, malfunctioning mitochondria, driven by increased fission and decreased fusion during sepsis. In septic models, it has been demonstrated that pharmacological therapies that either increase mitochondrial biogenesis or autophagy or decrease excessive mitochondrial fission improve mitochondrial function and lessen organ failure [49,50,51].

##### The Biogenesis of Mitochondria

The production of mitochondrial proteins is known as “mitochondrial biogenesis.” These proteins can be encoded by mtDNA) which encodes proteins mostly engaged in the oxidative phosphorylation pathway, or by nuclear DNA, which is then imported and incorporated into the mitochondria. This process improves the mitochondria’s capacity to generate energy and aids in the replacement of damaged proteins, especially as energy demands increase [52]. Thus, meeting the energy needs of cellular metabolism depends heavily on mitochondrial biogenesis.

##### The Process of Mitophagy

Dysfunctional mitochondria are eliminated and broken down via mitophagy, a type of selective autophagy. By recycling outdated mitochondrial components, it controls mitochondrial renewal and repair. By maintaining energy substrates, removing damaged mitochondria that can result in excessive ATP synthesis, and lowering oxidative stress, this mechanism also shields the heart from cell death. Inhibiting mitophagy can therefore lead to decreased mitochondrial mass, the buildup of defective mitochondria, and worsened sepsis outcomes [45,53,54]. To improve heart function, mitophagy is being investigated as a potential therapeutic target.

#### 2.3.3. Myocardial Depression Directly

A decrease in β-adrenergic receptor components causes the adrenergic response to be disrupted at the cardiomyocyte level, which is the initial molecular mechanism underlying direct cardiac depression. Numerous pro-inflammatory chemicals, mainly cytokines and nitric oxide (NO), influence this impact. Toxins, complements, DAMPs, and other myocardial depressants can cause cardiomyocyte damage and/or death, which is the second pathway [55]. Third, one of the main causes of myocardial depression and various organ failures associated with sepsis is cardiomyocyte apoptosis. If left untreated, cardiac failure can progress to cardiomyocyte apoptosis, which causes β-adrenoceptors to be downregulated and myofibril function to be compromised by disturbed calcium release.

##### The Cytokines

In response to stress or conditions such as surgery, trauma, ischemia, or sepsis, both somatic cells (such as endothelial, epithelial, and fibroblast cells) and immune cells (such as neutrophils, lymphocytes, and macrophages) produce cytokines locally. Cells exposed to these stressors can communicate more easily through these signals [56]. Additionally, prostanoids and nitric oxide (NO), two more inflammatory substances that exacerbate myocardial dysfunction, are released in response to cytokines. Among the most well-known pro-inflammatory cytokines released by macrophages during sepsis are tumor necrosis factor (TNF)-α and interleukin (IL)-1β, which have been demonstrated to dramatically reduce cardiac contractility in vitro [57]. Nitric oxide (NO) and oxygen-free radicals are thought to be secondary causes of septic myocardium depression, but TNF-α and IL-1β are acknowledged as important mediators of the inflammatory response [58,59]. IL-1, which is produced in response to TNF-α, stimulates nitric oxide synthase (NOS), which lowers cardiac contractility. Therefore, IL-1 inhibitors, such IL-1 receptor antagonists, may be a viable way to lower morbidity and increase survival in SIMD patients. But as of now, there is not enough data to fully support this strategy [60,61]. Myocardial dysfunction is worsened and complicated by elevated cytokine levels, which also stimulate the production of additional cytokines and chemical mediators. Furthermore, during inflammatory reactions, the heart itself may release cytokines, exacerbating myocardial depression and compromising cardiac function. This was especially noticeable when the heart released IL-6 due to excessive catecholamine use and activation of myocardial α- and β-adrenoreceptors. In addition to direct inflammatory and mitochondrial mechanisms, severe cytokine-driven inflammatory states may contribute to myocardial dysfunction through capillary leak and myocardial edema. Elevated interleukin levels—particularly IL-6 and IL-1β—promote endothelial activation and increased vascular permeability, resulting in interstitial fluid accumulation and altered ventricular loading conditions [62,63].

##### Nitric Oxide

The vascular endothelium produces NO, which has a wide range of physiological effects on the cardiovascular system and may be a mediator of SIMD. Different isoforms of nitric oxide synthase (NOS), which are found in subcellular compartments, produce NO, which functions as a second messenger in these components. While NOS isoform 2 has been linked to contractile depression during late sepsis, NOS isoforms 1 and 3 have been linked to early SIMD [64,65].

Nitric oxide (NO) has been connected in a number of studies to the degree of myocardial dysfunction and a higher death rate. This link results from NO’s effects on many sites, such as mitochondrial activity and the heart’s β-adrenergic receptors. One important mechanism in the development of SIMD is the impairment of mitochondrial activity, which is caused by NO [66].

##### Endothelin-1

Inflammatory cytokines have been demonstrated to be stimulated by elevated endothelin-1 (ET-1). Bosentan-induced endothelin-receptor blockage improved heart function in a pig endotoxic model [67]. Nevertheless, it is unclear how ET-1 contributes to septic cardiac dysfunction, which emphasizes the need for more study to determine how it works.

##### Intracellular Adhesion Molecules

Intercellular adhesion molecule-1 (ICAM-1) and vascular cell adhesion molecule-1 (VCAM 1) were expressed more in the coronary endothelium and cardiomyocytes of animals stimulated with LPS and TNF-α. Myocardial dysfunction was avoided and neutrophil buildup in the myocardium was decreased by blocking VCAM-1 [68,69]. Furthermore, although they had no effect on neutrophil accumulation, ICAM-1 deletion and antibody blocking both enhanced myocardial function during endotoxemia. Even while ICAM-1 or VCAM-1 antibody blockage can resolve contractile dysfunction, more research is required to comprehend the ways in which these adhesion molecules affect calcium homeostasis and the generation of oxygen-free radicals.

##### Prostanoids

Serum concentrations of prostacyclin, thromboxane, and other prostanoids are higher in septic patients. Cyclooxygenase inhibitors such as indomethacin can mitigate the possible disruption of coronary endothelial function caused by these prostanoids. Although clinical trials have demonstrated that neither medication significantly increases survival rates, the use of nonsteroidal anti-inflammatory medications (NSAIDs) such ibuprofen and lornoxicam to block prostanoids has been proposed as a therapy option [70].

##### High Mobility Group Box 1 (HMGB1) and Histones

On a variety of cell types, including cardiomyocytes, extracellular histones function as endogenous damage-associated molecular patterns (DAMPs) that may interact with TLR2 and TLR4. Cell damage and organ failure, including heart disease, can arise from this interaction, which can lower ATP levels and mitochondrial membrane potential. In sepsis patients, Alhamdi et al. discovered a correlation between elevated levels of circulating histones and elevated levels of cardiac troponin T (cTnT), which likely leads to septic cardiac dysfunction and death [55,71]. The development of cardiac dysfunction is significantly influenced by the pro-inflammatory mediator HMGB1. Zhang et al. showed that an increase in intracellular ROS levels brought on by the HMGB1–TLR4 interaction is one way that HMGB1 causes cardiac dysfunction. Increased oxidative stress results from this interaction [72]. Moreover, cardiac excitation–contraction (EC) coupling is disrupted by HMGB1. Myocyte contractility and systolic Ca^2+^ transients are thereby reduced. In conclusion, cardiac dysfunction results from the direct injury of myocytes or the release of damage-associated molecular patterns (DAMPs) by injured myocytes, such as histone and/or HMGB1.

##### Complement System

The complement system is triggered in sepsis, and multiorgan failure is closely linked to complement component 5 (C5a). The modification of calcium and ROS levels in cardiomyocytes, which results in cardiac dysfunction, is the reason for C5a’s participation in septic myocardial dysfunction [73]. Moreover, C5a-induced cardiodepression is mediated via cardiomyocyte expression of the C5a receptor (C5aR) [74].

##### Additional Mediators

Numerous novel endogenous molecules are being identified as possible causes of septic myocardium depression, and the evidence for more myocardial depressive drugs is continually growing. These consist of estrogenic chemicals, prostaglandins, histamine, and leukocyte lysozyme. But several recently discovered compounds, including endotoxin and natriuretic peptides, need more research. In comparison to controls, septic patients had considerably increased levels of both B-type natriuretic peptide and atrial natriuretic peptide. Although further research is required to completely understand these consequences, caspase-3 activation has been connected to structural breakdown of sarcomeres and decreased sensitivity of myofilaments to calcium. Furthermore, new information indicates that leukotrienes, protein kinase C, lipoteichoic acid, platelet-activating factor, cyclooxygenase products, and others may also contribute to septic myocardial depression [75,76,77].

#### 2.3.4. Epigenetic Mechanisms

In addition to inflammatory and metabolic signaling, epigenetic mechanisms appear to contribute to the pathogenesis of SIMD. Epigenetic regulation—including microRNAs (miRNAs), long non-coding RNAs (lncRNAs), DNA methylation, and histone modifications—modulates gene expression without altering the DNA sequence and influences inflammatory signaling, oxidative stress, mitochondrial function, and cardiomyocyte survival in the septic heart. Several miRNAs have been implicated in SIMD: miR-146a and miR-21 regulate NF-κB-mediated inflammatory responses, whereas miR-155 has been associated with enhanced inflammation and myocardial depression. Additionally, miR-223 and miR-499 appear to modulate apoptosis and mitochondrial integrity in cardiomyocytes. These emerging epigenetic mechanisms broaden the current understanding of septic myocardial dysfunction and may represent potential targets for biomarker development and therapeutic intervention [78,79].

## 3. Discussion

### 3.1. Monitoring Strategies

To effectively measure the effects on heart function, monitoring sepsis-induced cardiac dysfunction requires a multimodal approach. Important components consist of:

1. Hemodynamic monitoring: Regular evaluation of vital signs, such as blood pressure, heart rate, and central venous pressure, aids in determining the general state of the cardiovascular system. Advanced hemodynamic monitoring tools, like pulmonary artery catheters and the transpulmonary thermodilution method, offer comprehensive data on cardiac output, LV ejection fraction, preload, and afterload. They also assist in assessing the efficacy of fluid resuscitation and the sufficiency of perfusion. In cases of sepsis-induced myocardial dysfunction, measuring cardiac output (CO) and other hemodynamic parameters is essential. Although the pulmonary artery catheter (PAC) was once often used to monitor hemodynamics in critically ill patients, its use has decreased because there is no proof that it reduces patient mortality. Another technique for measuring CO and cardiac performance indicators such as the global ejection fraction (GEF) and cardiac function index (CFI) is the transpulmonary thermodilution method. According to reports, cardiac dysfunction in septic patients can be detected by low CFI and GEF readings from transpulmonary thermodilution. Furthermore, it has been determined that pulse contour analysis is a reliable method for continuous CO assessment in sepsis. Nevertheless, more research is needed to confirm these techniques’ effectiveness in identifying SIMD [80,81,82]. There is evidence that non-invasive hemodynamic techniques, such as end-expiratory occlusion, passive straight leg raise, inferior vena cava collapse, pulse contour analysis, pulse pressure variation, and stroke volume fluctuation, are effective in determining fluid responsiveness in septic shock patients. These methods do have certain drawbacks, though. Following the first stage of resuscitation, cautious fluid control is crucial. Dynamic measurements of fluid responsiveness ought to serve as guidance for this.
2. Echocardiography: This imaging modality provides real-time assessment of cardiac anatomy and function, enabling the evaluation of ventricular size, ejection fraction, and regional wall motion abnormalities—key features of SIMD. In addition, continuous electrocardiographic monitoring is essential for the detection of ischemic changes or arrhythmias that may arise secondary to sepsis-related cardiac dysfunction.
3. Cardiac Blood Biomarkers: Cardiac troponins and B-type natriuretic peptide (BNP) are widely used biomarkers for assessing myocardial injury and wall stress. Elevated concentrations of these markers are associated with cardiac dysfunction and may aid in risk stratification and therapeutic decision-making.
4. Lactate levels: Serial monitoring of serum lactate levels assists in the assessment of tissue perfusion and oxygenation. Elevated lactate concentrations may indicate inadequate cardiac output and can inform timely adjustments in therapeutic management.
5. Clinical assessment: Frequent examination of clinical indicators, such as edema, jugular venous distension, and altered mental status, can reveal information concerning declining fluid balance and cardiac function. Integrating these data points is necessary for effective monitoring in order to give a thorough picture of heart function and direct suitable therapeutic techniques for sepsis patients.

### 3.2. Differential Diagnosis of Myocardial Dysfunction in Septic Patients

Myocardial dysfunction observed during septic shock frequently overlaps with other acute cardiac conditions. Stress-related cardiomyopathy triggered by severe infection or cytokine storm may closely resemble SIMD both clinically and echocardiographically. However, Takotsubo syndrome typically demonstrates left ventricular apical ballooning whereas SIMD more often presents with global or biventricular systolic impairment. Acute myocarditis should be suspected when there is disproportionate troponin elevation or persistent dysfunction despite resolution of septic shock. In such cases, cardiac magnetic resonance (CMR) may reveal myocardial edema and late gadolinium enhancement consistent with inflammatory injury. Last, type 2 myocardial infarction, driven by oxygen supply–demand imbalance, may also contribute to ventricular dysfunction in septic patients, particularly in those with underlying coronary artery disease. Clinical context, dynamic ECG changes, and the pattern of troponin elevation may aid differentiation. Furthermore, decompensation of pre-existing cardiomyopathy should be considered, especially in patients with prior structural heart disease.

### 3.3. Handling Myocardial Dysfunction

Management of sepsis-induced myocardial dysfunction should prioritize prompt control of the underlying infection, in addition to supportive measures such as fluid resuscitation, oxygen therapy, and pharmacological support aimed at restoring cardiac function. Early recognition, source control, and optimization of hemodynamic status through appropriate fluid resuscitation and vasopressor therapy constitute the cornerstone of initial sepsis management and are considered the standard of care for SIMD. Recent updates from the Surviving Sepsis Campaign 2021 guidelines further emphasize early administration of broad-spectrum antimicrobials, balanced crystalloid resuscitation, and individualized hemodynamic support guided by dynamic assessment of fluid responsiveness [2]. Fluid resuscitation plays a critical role by increasing cardiac output, thereby improving oxygen delivery, organ perfusion, and reversing sepsis-related hypoperfusion. However, excessive fluid amounts following first resuscitation may result in tissue edema and a rise in heart filling pressure, both of which are associated with increased death rates [81,82]. “Hemodynamic monitoring” is advised by current worldwide standards for the management of sepsis. Therefore, to evaluate fluid responsiveness and direct fluid therapy, the hemodynamic status should be regularly evaluated. LV end-diastolic area by echocardiography, global end-diastolic volume by a transpulmonary thermodilution, pulse pressure variation and systolic pressure variation by arterial waveform, stroke volume variation by pulse contour analysis, and central venous pressure by central venous catheter are methods to assess fluid responsiveness [83,84,85]. The recommended vasopressor for treating sepsis patients is norepinephrine, which functions as both an alpha and beta agonist. Although there is not enough solid evidence to support its use, current guidelines support the use of dobutamine in the context of myocardial dysfunction, such as increased filling pressures, reduced cardiac output, or symptoms of prolonged hypoperfusion [84].

In sepsis and SIMD, levosimendan, an inotropic calcium sensitizer, has been demonstrated to improve cardiac output and systemic hemodynamics with a negligible increase in oxygen consumption [86]. However, in their meta-analysis, Liu et al. [87] showed that levosimendan usage had no influence on mortality. When tachycardia makes it difficult to maintain blood pressure and systemic circulation, beta-blockers may be useful. However, neither the 28-day mortality nor the SOFA (Sequential Organ Failure Assessment) score improved in recent research assessing the effects of landiolol in patients with sepsis and tachycardia when taken with norepinephrine [6,88].

The limited impact of agents such as levosimendan and landiolol on mortality may reflect a fundamental mismatch between hemodynamic modulation and the underlying molecular drivers of SIMD. Although levosimendan enhances calcium sensitivity and improves contractility, it does not directly correct mitochondrial energetic failure or reverse oxidative damage, which are central to septic myocardial depression. Similarly, beta-blockade with landiolol may attenuate excessive adrenergic stimulation and improve heart rate control, yet it does not address impaired ATP production, altered mitochondrial dynamics, or regulated inflammatory cell death pathways.

If conservative treatment is ineffective for sepsis-related cardiogenic shock, mechanical support such as an intra-aortic balloon pump (IABP), venoarterial ECMO (VA-ECMO), or an Impella device that can improve cardiac output is deemed necessary [89].

Nonetheless, the use of mechanical circulatory support in septic shock is further complicated by the complex hematologic and vascular characteristics of severe sepsis. Thrombocytopenia, sepsis-associated coagulopathy, disseminated intravascular coagulation, and increased bleeding risk frequently limit the safe implementation of extracorporeal devices. In addition, profound vasoplegia and altered vascular tone may reduce the effectiveness of certain mechanical strategies by impairing ventriculo-arterial coupling and systemic perfusion despite adequate mechanical flow. Therefore, escalation to mechanical circulatory support should be guided by careful hemodynamic phenotyping using advanced monitoring techniques, including echocardiography, transpulmonary thermodilution, and, when available, assessment of ventriculo-arterial coupling

Various pharmacological agents have been investigated for the treatment of sepsis with the aim of preventing SIMD and multiorgan dysfunction; however, these therapies have not resulted in substantial improvements in clinical outcomes. This highlights the need for novel therapeutic strategies that specifically target the underlying mechanisms of sepsis-related cardiac dysfunction.

### 3.4. Emerging and Targeted Therapeutic Approaches

Beyond conventional hemodynamic support, increasing attention has been directed toward mechanism-based therapies targeting the molecular drivers of SIMD. Given the central role of mitochondrial injury, oxidative stress, and dysregulated inflammation, several experimental strategies are under investigation. Agents such as mitochondria-directed antioxidants (e.g., MitoQ, elamipretide) have shown cardioprotective effects in preclinical models by improving mitochondrial respiration and limiting oxidative damage. Strategies enhancing mitochondrial biogenesis or modulating mitophagy are also being explored. In parallel, inhibition of ferroptosis and other forms of regulated cell death has emerged as a potential approach to attenuate cardiomyocyte loss in septic cardiomyopathy. Moreover, targeted immunomodulation represents another promising direction. Although broad anti-inflammatory therapies have largely failed in clinical trials, selective blockade of specific mediators—such as interleukin-1 or complement component C5a—has shown potential in experimental studies. In addition, inhibition of necroptosis signaling pathways may reduce inflammatory myocardial injury [45,73]

## 4. Materials and Methods

In the present review, we performed a literature search in PubMed and Scopus databases by using ‘myocardial dysfunction’, in combination with ‘sepsis’ and ‘septic shock’, between 1990 and 2025. We included relevant articles and reviews regarding the Association between myocardial dysfunction and septic shock. Exclusion criteria were as follows: studies written in languages other than English; letters to the editor; conference presentations; editorials; comments; opinions; and articles without free access.

## 5. Conclusions

The pathophysiology and molecular mechanisms underlying sepsis-induced cardiac dysfunction remain incompletely understood. Despite decades of intensive research, effective treatment of sepsis-related myocardial dysfunction continues to be a major clinical challenge. Current sepsis management relies on a multimodal supportive approach—including infection control, antimicrobial therapy, fluid resuscitation, vasopressors, inotropes, and comprehensive hemodynamic monitoring—yet evidence supporting targeted cardiac-specific therapies remains limited. Although numerous clinical and experimental studies have explored therapeutic strategies for sepsis and its cardiovascular manifestations, many have yielded inconclusive or negative results upon validation. Consequently, the development of more effective treatment strategies depends on a deeper understanding of the biological mechanisms driving sepsis-induced cardiac failure.

## Data Availability

No new data were created or analyzed in this study. Data sharing is not applicable to this article.
